# Size Prediction and Electrical Performance of Knitted Strain Sensors

**DOI:** 10.3390/polym14122354

**Published:** 2022-06-10

**Authors:** Xinhua Liang, Honglian Cong, Zhijia Dong, Gaoming Jiang

**Affiliations:** Engineering Research Center for Knitting Technology, Ministry of Education, Jiangnan University, Wuxi 214122, China; liangxh18861852560@163.com (X.L.); dongzj0921@163.com (Z.D.); jgm@jiangnan.edu.cn (G.J.)

**Keywords:** knitted fabrics, strain sensors, size prediction, precise positioning, human motion detection

## Abstract

Benefitting from the multifunctional properties of knitted fabrics with elasticity, flexibility, and high resilience, knitted strain sensors based on structure and strain performance are widely utilized in sports health due to their adaptability to human movements. However, the fabrication process of common strain sensors mainly relies on experienced technicians to determine the best sensor size through repeated experiments, resulting in significant size errors and a long development cycle. Herein, knitted strain sensors based on plain knit were fabricated with nylon/spandex composite yarn and silver-plated nylon yarn using a flat knitting process. A size prediction model of knitted strain sensors was established by exploring the linear relationship between the conductive area size of samples and knitting parameters via SPSS regression analysis. Combined with stable structures and high performance of good sensitivity, stability, and durability, the knitted strain sensors based on size prediction models can be worn on human skin or garments to monitor different movements, such as pronunciation and joint bending. This research indicated that the reasonable size control of the knitted strain sensor could realize its precise positioning in intelligent garments, exhibiting promising potential in intelligent wearable electronics.

## 1. Introduction

With the foreseeable prosperity and integration of medical electrical devices, textile equipment, and human health monitoring equipment, wearable devices have attracted considerable attention from investigators due to their promising applications in human motion detection, soft robotics, electronic skins, and sensors [1,2,3,4,5,6,7]. Knitted strain sensors with the attributes of being lightweight, having good flexibility, and with a wide strain range have been emerging, promising a myriad of applications in the development of wearable sensing devices [8,9,10,11,12,13]. So far, plenty of knitted strain sensors are coated with conductive materials, such as graphene [14,15,16], polypyrrole [17,18], PEDOT: PSS [19], and nano-silver [20,21], showcasing the advantages of good stability, high sensitivity, and fast response. Various novel fabrication methods [22,23,24] have been proposed to knit the conductive yarn into the conductive fabric directly, forming an intelligent wearable device with sensing performance. However, these types of sensors are structurally unstable and have a small strain range, which definitely impedes their practical application [25,26].

In addition, yarn type, structure, and the interaction between loops are also important factors affecting the knitted sensor’s performance. Production parameters, such as structural changes, spandex content, and the washing and ironing processes, play a fundamental role in determining the sensors’ physical behavior and sensing performance [27,28,29]. Most strain sensors based on knitted structures rely on resistance changes [30,31,32]. For instance, Liu et al. proposed a geometric model incorporated with a simplified resistive network, determining the resistance effect of conductive float stitches on knitted structures with different courses and wales [33]. To evaluate the overall degree of plane anisotropy of knitted fabrics, a new measurement procedure was established using the van der Pauw electrode configuration to solve the issue of measuring the edge resistance of fabrics [34]. Knitting elements with different proportions also have a considerable impact on the conductive fabric resistance [35]. Li et al. produced a resistance model of conductive knitted fabric under unidirectional stretching, superimposing length resistance and contact resistance to simulate the fabric resistance [36]. The as-developed resistance calculation systems reveal the discipline of resistance variation in conductive knitted fabrics, offering a theoretical reference in conductive fabric design.

The size prediction of knitted fabric is a considerable segment in the garment design and production process. For example, Liu et al. developed the size prediction model of warp-knitted Jacquard fabric to investigate the relationship between the yarn count, tensile density, and the size shrinkage rate, utilizing JavaScript and WebGL technologies to automatically generate clothing templates [37]. A structural model correlating the size of tubular knitted fabric with the loop geometric parameters was produced, deducing yarn-feeding parameters according to the elasticity and size requirements of the fabric [38]. Ulson et al. put forward a prediction system of circular-knitted cotton fabric, introducing an approach that saves time and money while improving knitted fabric quality for customers [39].

To date, the study on knitted strain sensors has mainly focused on the influence of elements of resistance, the resistance calculation model, and the sensor design and application, whereas there are few researches on size control and prediction. Intelligent garments monitoring human movements require that sensors be able to respond instantly and sensitively to changes in human joints and skin. In the actual development process, samples should be designed at least 2–3 times to determine the sensors’ sizes at different parts of the human body, and the finishing process is also more complicated, resulting in a significant increase in cost and product losses. This makes the size control the focus and nodus in the design and fabrication of knitted strain sensors.

Herein, knitted strain sensors based on plain knit were successfully fabricated with nylon/spandex composite yarn and silver-plated nylon yarn using a flat knitting process. By investigating the influence of different knitting parameters on a sensor’s size, the size prediction model was established. To assess its potential to serve as a strain sensor, electrical performance tests were carried out to probe its sensitivity, hysteresis deviation, working sense range, and repeatability under various stretching rates. Furthermore, the as-prepared strain sensor based on the size prediction model was applied to monitor different human movements in order to verify its accuracy, indicating its promising potential for use in intelligent wearable devices.

## 2. Experimental Methods

### 2.1. Materials

In this experiment, silver-plated nylon yarn (222dtex/48F, the electrical resistance is 6.5 Ω/cm) was purchased from Qingdao Hengtong Weiye Special Fabric Technology Co., Ltd. (Qingdao, China). Nylon/spandex composite yarns (22/55dtex, 22/77dtex, and 44/77dtex) were purchased from Hubei Yutao Special Fiber Co., Ltd. (Chongyang, China). Elastic nylon filament (333dtex/24F) was obtained from Jiangsu Pingmei Yarn Industry Co., Ltd. (Hai’an, China).

### 2.2. Preparation of Knitted Strain Sensors

**Figure 1:**
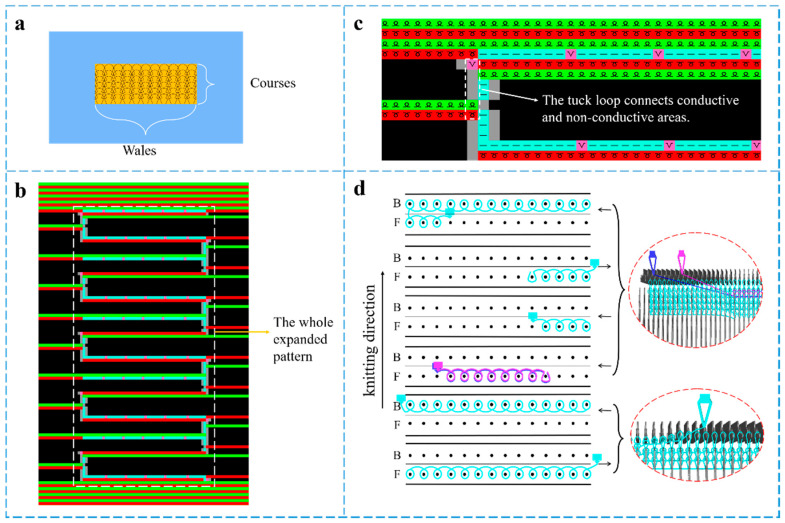
Pattern design and knitting process of the knitted strain sensor (**a**) compression pattern design (**b**) expanded pattern in the design system and (**c**) single pattern cycle (**d**) the knitting process and simulation of a single pattern.

The plain knit structure has been proved to be a promising candidate as the fabric substrate due to its merits of compact structure, considerable flexibility, and relatively excellent electrical performance [40]. Figure 1 exhibits the pattern design and knitting process of the strain sensors. The machine parameters of a sensor’s compression pattern transformed into an expanded pattern via automatic control instructions were set in SDS-ONE APEX3 pattern design system matching with a computerized flat knitting machine. Three yarn feeders were used in the knitting process, working from bottom to top.

**Figure 2:**
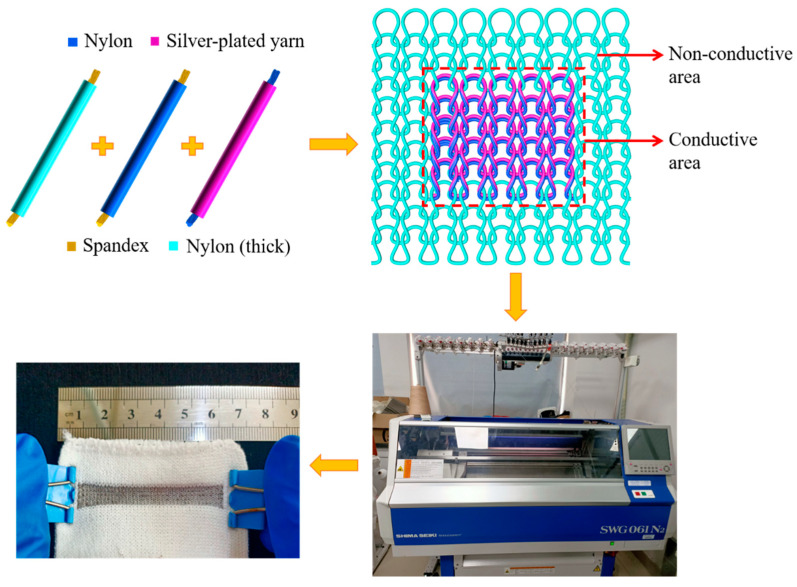
The fabrication process of the knitted strain sensor.

The preparation process of the knitted strain sensor is illustrated in Figure 2. The elastic nylon filament was knitted to form the non-conductive area as a fabric substrate. Every loop in the conductive area consisted of two overlapping yarns, in which the silver-plated nylon yarn appeared at the technical surface of the strain sensors, with the nylon/spandex composite yarn at the back. The two areas were connected by a tuck loop.

Considering the feasibility of knitting, the measurability, and the reduction in excess waste, the wales of the sensor’s conductive area were set as 30, 40, and 50, and the courses were set as 10, 20, and 30, respectively. The specifications of all samples are shown in Table 1, where EN, SN, and NS are elastic nylon filament, silver-plated nylon yarn, and nylon/spandex composite yarns, respectively.

### 2.3. Characterization and Measurements

#### 2.3.1. Size-Change Test

Under the ironing condition of 150 °C and 0.4 MPa, the knitted strain sensors were ironed with a BOG energy-saving steam generator (Yancheng Xuanlang Machinery Equipment Co., Ltd., Yancheng, China). The size-change principle of the sensors was analyzed by SPSS regression analysis.

#### 2.3.2. Electrical Performance Test

The electrical performance of a sensor largely depends on its strain. When the sensor is stretched, the contact conditions between the loops change, resulting in corresponding changes in its resistance [41]. In this paper, a sensor’s resistance from 0−100% strain range was measured. The strain was implemented to the sensor by the electric stretching/compression test bench (Beijing Jipin Times Technology Co., Ltd., Beijing, China). The resistance was recorded using a CHI760 electrochemical analyzer (Shanghai Chenhua Instrument Co., Ltd., Shanghai, China), which presents the corresponding time-current (I-T) characteristic curve.

## 3. Results and Discussions

### 3.1. Relationship between Knitting Factors and Sensor Size

The horizontal and vertical sizes of the conductive area in the sensors with different knitting factors were measured, including the gray and the finished samples. Size shrinkage of the samples was calculated by Formulas (1) and (2).

(1) $$S_{h}=\left(X_{h}-Y_{h}\right)/X_{h}\times 100\%,$$

(2) $$S_{v}=\left(X_{v}-Y_{v}\right)/X_{v}\times 100\%,$$

where $X_{h}$ and $Y_{h}$ represent the horizontal size of the gray and the finished samples, respectively. $S_{h}$ represents the horizontal size shrinkage. $X_{v}$ and $Y_{v}$ represent the vertical size of the gray and the finished samples, respectively. $S_{v}$ represents the vertical size shrinkage.

#### 3.1.1. The Effect of Knitting Factors on Sensors’ Horizontal Size

**Figure 3:**
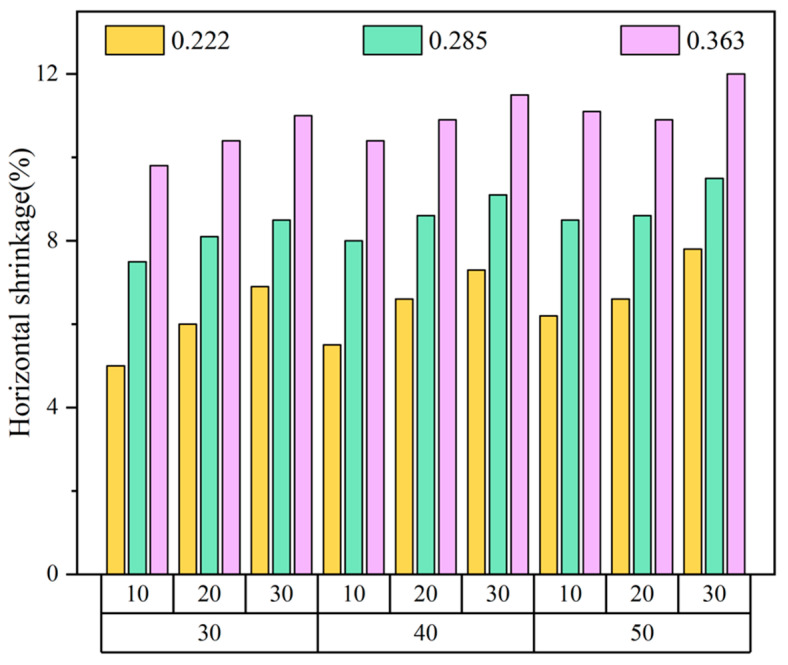
Change rate of sensors’ horizontal size.

Figure 3 reveals the change rate of the sensors’ horizontal size. When the wales and courses were constant, the horizontal change rate of the samples increased gradually with the increase of spandex content. There was inevitable residual stress in the nylon and spandex fibers, producing a shrinkage phenomenon when heated at a high temperature. A sensor with a high spandex content had a large shrinkage rate. It also increased with the augmentation of the wales and courses under the uniform yarn composition. This resulted mainly from the increase in the length of the needle and sinker loops, causing a higher shrinkage rate during the process.

#### 3.1.2. The Effect of Knitting Factors on Sensors’ Vertical Size

**Figure 4:**
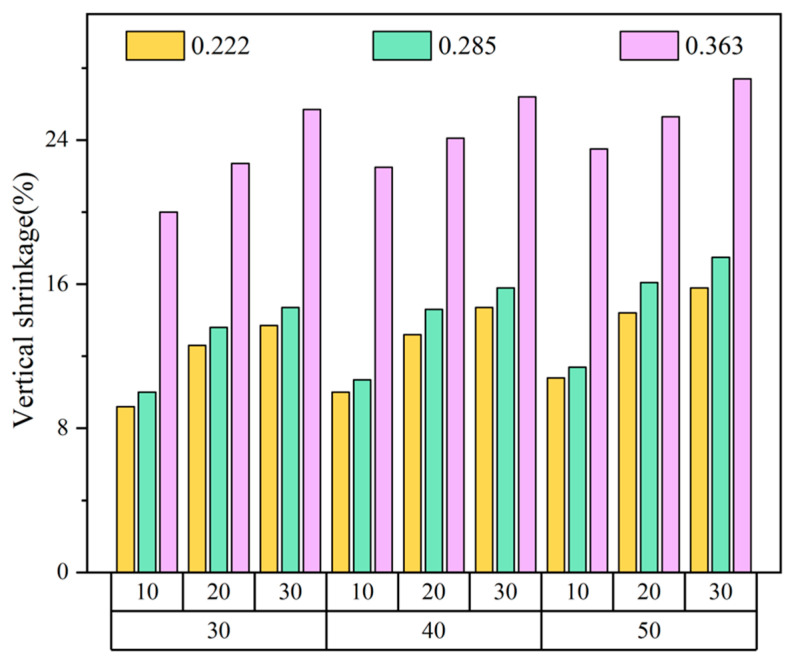
Change rate of sensors’ vertical size.

The change rate of the sensors’ vertical size, similar to that of the horizontal size, is shown in Figure 4. It increased with the addition of the spandex content, wales, and courses, which was mainly affected by the elastic shrinkage property of spandex and the number of leg yarn segments. The change rate of samples with vertical size exceeded that of those of horizontal size. The probable explanation is that the length of the needle and the sinker loop is shorter than the leg yarn segment in a loop with a compact arrangement. Therefore, the length change of the latter surpasses the former when subjected to heat contraction, leading to a higher vertical shrinkage rate.

#### 3.1.3. Size Prediction Model of the Sensors

To probe the relationship between the spandex content, wales, courses, and sensor size, SPSS was utilized for regression analysis of the sorted data. S represents significance, which is the basis for judging whether R (correlation coefficient) is of statistical significance. The correlation coefficient between the two variables has no statistical significance when S > 0.05. The S and R values of the samples are shown in Table 2. The three knitting parameters are highly correlated with the size shrinkage of the sensors, and all have a significant effect.

In the light of the analysis by SPSS, the horizontal and vertical shrinkage rate of the sensor size was calculated using the following Formulas (3) and (4), respectively.

(3) $$S_{h}=-3.617+0.044x+0.064y+31.567z,$$

(4) $$S_{v}=-37.552+0.101x+0.215y+83.334z,$$

where x represents the wales, y represents the courses, and z represents the spandex content. It can be observed from the equation that the dependent coefficients of the horizontal and vertical shrinkage rates are all positive, indicating that the size shrinkage in two directions of the samples increases with the three parameters. In combination with Formulas (1) and (2), the calculation methods of the horizontal and vertical sizes of the sensors can be described as Formulas (5) and (6), respectively.

(5) $$Y_{h}=\frac{x}{P_{x}}-\frac{\left(-3.617+0.044x+0.064y+31.567z\right)\cdot P_{x}}{x},$$

(6) $$Y_{v}=\frac{y}{P_{y}}-\frac{\left(-37.552+0.101x+0.215y+83.334z\right)\cdot P_{y}}{y},$$

In conclusion, the size prediction and control of the sensors can be realized by changing the number of wales, courses, and the spandex content, providing a certain guiding significance for future research.

### 3.2. Electrical Performance of the Knitted Strain Sensor

**Figure 5:**
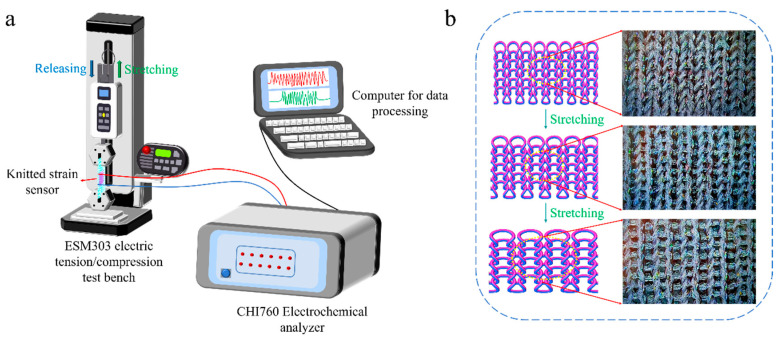
Research on sensing mechanism of the knitted strain sensor, (**a**) illustration of the measuring device, and (**b**) loop change of the strain sensor during the horizontal stretching process.

To illustrate the electrical properties of the knitted strain sensor, the sensing mechanism was systematically explored by simulating the relationship between resistance and deformation under the stretching–releasing process. As shown in Figure 5a, the knitting strain sensor connected to the CHI760 electrochemical analyzer through two collets (red wire and blue wire) was held at both ends of the ESM303 electric stretching/compression test bench along the horizontal direction. The sensor was stretched 0–100% at the stretching rate of 100 mm min^−1^. Furthermore, a computer was affiliated to the CHI760 electrochemical analyzer through a multifunctional communication cable (black wire) to output the current change with time during the stretching–releasing process in real time. In the data processing software matched with the electrochemical analyzer, the applied AC voltage was set to 0.1 V. Then, the current data was processed by Excel software according to the formula: R = V/I, and the corresponding data of resistance change with time was obtained.

The loop structure of the knitted strain sensor can be regarded as a complex series and parallel electrical network involving the resistance of each yarn segment and contact resistance [42]. Figure 5b expounds on the loop changes of the sensor during the cycle test. The contact resistance change played a governing function in the horizontal stretching of the fabric, whereas the resistance change caused by loop transfer had little effect on the sensor’s resistance. In the initial stage of stretching, the leg yarn segments transferred to the needle and sinker loops [43], and the number of contact points changed rapidly, accounting for the higher sensitivity of the sensor. With the further increase in external stretching, the transfer of the yarn segment was no longer evident, resulting in a smaller change in the number of contact points. Herein, lower sensitivity was observed for higher stretching.

Sensitivity is one of the critical parameters in determining a sensor’s performance. It usually represents the ratio of sensor output to input, elaborating the accuracy and effectiveness of the sensor [44]. GF is traditionally described as the gauge factor, which is defined as the following Formula (7).

(7) $$GF=\frac{\left(R-R_{0}\right)/R_{0}}{\varepsilon }=\frac{\Delta R/R_{0}}{\varepsilon },$$

where *R*_0_ is the initial resistance, *R* refers to the real-time resistance of the sensor in the stretching–releasing process, and *ε* represents the strain applied to the sensor.

**Figure 6:**
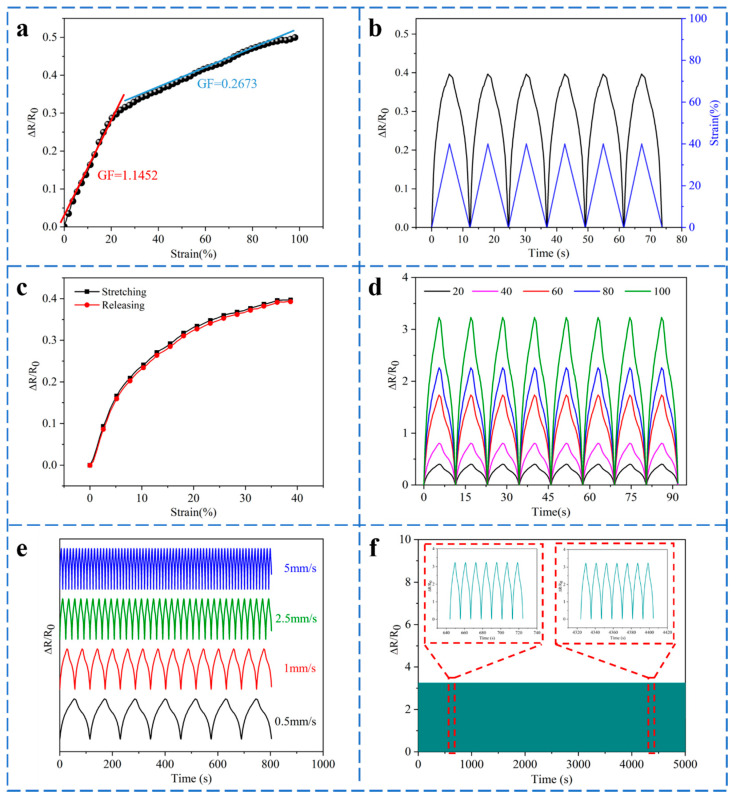
Sensing performance of the knitted strain sensor, (**a**) the ∆*R*/*R*_0_ curve of the sensor with strain, (**b**) the curves of ∆*R*/*R*_0_ and strain varying with time, (**c**) single stretching–releasing curve of the sensor, (**d**) the curves of ∆*R*/*R*_0_ under different strains (0–100%), (**e**) ∆*R*/*R*_0_ of strain sensor under different stretching rates of 40% strain, (**f**) the stretching–releasing tests under a cyclic strain of 100%.

As depicted in Figure 6a, the variation of ∆*R*/*R*_0_ with strain in the horizontal stretching of the sensor can generally be divided into two phases. The ∆*R*/*R*_0_ of the sensor shows a rapid upward trend in phase I when the strain <25% (GF = 1.1452), whereas it slows down and gradually becomes gentle in phase II in the strain range 25–100% (GF = 0.2673). This is consistent with the fabric-sensing mechanism described in Figure 5b. The more significant the change of contact points during stretching, the higher the sensitivity.

Figure 6b reveals the variation curves of ∆*R*/*R*_0_ and strain with time, which change almost synchronously, indicating a good response of the knitted strain sensor to the applied strain. The signal is from F1 for the following electrical performance test. The stretching–releasing curves of the sensor are revealed in Figure 6c. They almost overlap, although the two have a specific height difference. The maximum hysteresis of about 1.78% occurs when the strain is about 25%, indicating that the sensor has low hysteresis and good sensing performance. The elastic hysteresis and the energy absorption in the loops will affect the hysteresis of the sensor. Figure 6d reveals the dynamic response of the sensor under strain (0–100%). Evidently, the value of ∆*R*/*R*_0_ is practically identical under the same strain and increases gradually with the continuous augmentation of strain, demonstrating great repeatability of the sensor under different strains. Figure 6e declares the ∆*R*/*R*_0_ curve of the knitted strain sensor under four different stretching rates of 0.5, 1, 2.5, and 5 mm/s with a strain of 40%. The ∆*R*/*R*_0_ is highly consistent with the stretching curve and remains stable at each stretching rate. To illustrate the repeatability and durability of the sensor, 500 stretching–releasing cycles at 100% strain are shown in Figure 6f. It can be observed that the knitted strain sensor shows good repeatability and stability in electrical performance tests.

### 3.3. Application of the Size Prediction Model

**Figure 7:**
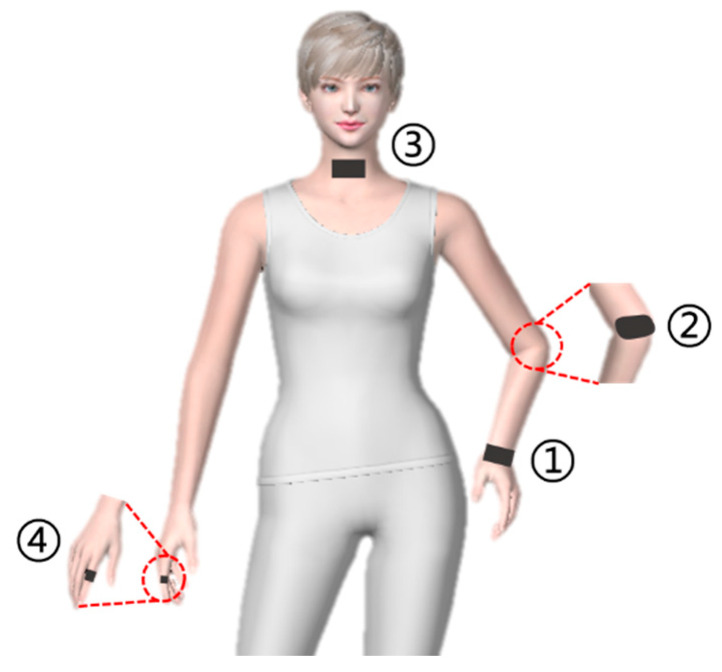
Different body parts for motion detection.

As illustrated in Figure 7, the as-designed knitted sensors under the size prediction model can be applied as a wearable device to four human body parts (wrist ①, elbow ②, throat ③, and forefinger ④) of a female volunteer to detect human movements. The horizontal and vertical sizes of different body parts are displayed in Table 3. Taking into account the effect of size changes and electrical properties, the parameters of the sensors’ conductive area based on nylon/spandex composite yarn with better elasticity (44/77dtex, z = 0.36) in different body parts are revealed in Table 4. The size deviation rates of the four knitted strain sensors are within the acceptable range of 5%, verifying the correctness of the size prediction model.

**Figure 8:**
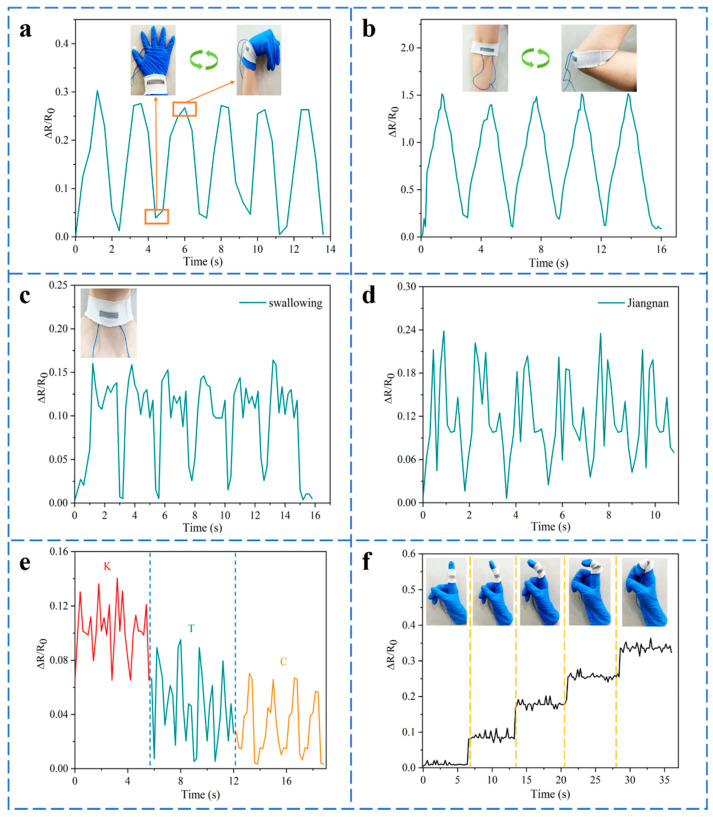
Application of the knitted strain sensors under the size prediction model in detecting different human movements, (**a**) wrist bending, (**b**) elbow bending, (**c**) swallowing of food, (**d**) speaking the word ‘Jiangnan’, (**e**) pronouncing words ‘K’, ‘T’, and ‘C’, (**f**) responsive curve of the sensor on the finger under diverse bending degrees.

The joint bending displayed in Figure 8a,b indicates that the sensor with a wide workable strain range can detect human movements. The ∆*R*/*R*_0_ of the sensor is consistent with the joint motions. In addition, the strain sensor can detect subtle movements. Figure 8c indicates that the sensor was attached to the throat with the continuous swallowing of food, and the ∆*R*/*R*_0_ of the sensor had a relatively consistent response. Pronunciation could also be discriminated by repeatedly reading the words ‘Jiangnan’, ‘K’, ‘T’, and ‘C’, as demonstrated in Figure 8d,e. To further probe the strain sensor’s capabilities in series motion detection, it was adhered to the forefinger to collect signals. As revealed in Figure 8f, the finger bent from small to large degrees. The resistance changed synchronously with the finger deformation when it bent, indicating a good discernible ability for subtle motion. All these indicate that the as-designed knitted strain sensor exhibits a potential application prospect in smart wearable devices in the near future.

## 4. Conclusions

In short, a knitted strain sensor of silver-plated nylon yarn and nylon/spandex composite yarn was successfully fabricated based on the knitting process. The numbers of wales, courses, and spandex content significantly affect the size of the knitted strain sensor. The change rate of samples with vertical size surpasses that of the horizontal size. The horizontal and vertical size prediction model of the knitted strain sensor was established. The sensor has a relatively good sensitivity of 1.1452 (strain ≤ 25%) with a rather large workable strain range (0–100%), good hysteresis, durability and stability over 500 cycles, ability to distinguish various tensile rates and strains, and good synchronization between output resistance and strains. The knitted strain sensors with different conductive areas based on the established size prediction model were designed and applied to human skin or clothing to monitor subtle and large-scale movements of the human body, such as pronunciation and joint bending, confirming the accuracy of the size prediction model. The results demonstrate that the sensor has a promising application prospect in intelligent wearable garments.

## Figures and Tables

**Table 1:** Knitting parameters of the experimental samples.

Fabric No.	Wales	Courses	Yarn Composition			Spandex Content (%)
			Non-Conductive Area	Conductive Area		
				Plating Yarn	Ground Yarn	
F1	30	10	EN	SN	20/50 NS	28.5
F2					20/70 NS	22.2
F3					40/70 NS	36.3
F4		20			20/50 NS	28.5
F5					20/70 NS	22.2
F6					40/70 NS	36.3
F7		30			20/50 NS	28.5
F8					20/70 NS	22.2
F9					40/70 NS	36.3
F10	40	10			20/50 NS	28.5
F11					20/70 NS	22.2
F12					40/70 NS	36.3
F13		20			20/50 NS	28.5
F14					20/70 NS	22.2
F15					40/70 NS	36.3
F16		30			20/50 NS	28.5
F17					20/70 NS	22.2
F18					40/70 NS	36.3
F19	50	10			20/50 NS	28.5
F20					20/70 NS	22.2
F21					40/70 NS	36.3
F22		20			20/50 NS	28.5
F23					20/70 NS	22.2
F24					40/70 NS	36.3
F25		30			20/50 NS	28.5
F26					20/70 NS	22.2
F27					40/70 NS	36.3

**Table 2:** The significance and correlation of knitting parameters with sensor size.

Knitting Parameters	Factors	The Size Shrinkage (%)	
		Horizontal Direction	Vertical Direction
Spandex content	S	0.000	0.000
	R	0.987	0.936
Wales	S	0.000	0.038
	R	0.973	0.861
Courses	S	0.000	0.000
	R	0.978	0.877

**Table 3:** Horizontal and vertical sizes of different body parts.

Body Part	Horizontal Size/cm	Vertical Size/cm
①	4.97	2.13
②	6.52	4.25
③	3.16	1.92
④	1.85	1.98

**Table 4:** The predicted and finished sizes of the sensors in different body parts.

Body Part	x	y	z	Predicted Size/cm		Finished Sizes/cm		Deviation Rate/%	
				Horizontal Direction	Vertical Direction	Horizontal Direction	Vertical Direction	Horizontal Direction	Vertical Direction
①	59	40	0.36	4.78	1.97	4.85	2.05	1.44	3.90
②	72	66	0.36	6.33	4.07	6.44	4.16	1.71	2.16
③	46	32	0.36	2.97	1.74	3.07	1.81	3.26	3.87
④	38	28	0.36	1.72	1.80	1.79	1.87	3.91	3.74

## Data Availability

The data presented in this study are available on request from the corresponding author.
